# Effectiveness of *Non-Presential Individualized Exercise Training PrOgram* *(NIETO)* in Lower Limb Physical Performance in Advanced COPD

**DOI:** 10.3390/jcm10051010

**Published:** 2021-03-02

**Authors:** Juan Miguel Sánchez-Nieto, Irene Fernández-Muñoz, Andrés Carrillo-Alcaraz, Roberto Bernabeu-Mora

**Affiliations:** 1 Division of Pneumology, Morales Meseguer General University Hospital, 30008 Murcia, Spain; juanm.sanchez3@carm.es (J.M.S.-N.); irene.fernandez@ffis.es (I.F.-M.); 2 Institute for Bio-Health Research of Murcia (IMIB-Arrixaca), El Palmar, 30120 Murcia, Spain; baypap@movistar.es; 3 Department of Internal Medicine, University of Murcia, El Palmar, 30120 Murcia, Spain; 4 Division of Intensive Care Unit, Morales Meseguer General University Hospital, 30008 Murcia, Spain

**Keywords:** COPD, physical activity, pulmonary rehabilitation, exercise training, non-presential program, quadriceps muscle strength, four-meters gait-speed test, five-repetition sit-to-stand test, Short Physical Performance Battery, six-minute-walk test

## Abstract

Muscle training, a component of pulmonary rehabilitation (PR), improves the physical performance of patients with chronic obstructive pulmonary disease (COPD). Despite the existing evidence, the traditional center-based PR model is applied to a small percentage of patients and presents numerous problems of accessibility, adherence, and costs. This study presents a home model of simple muscle training, non-presential, monitored by telephone and individualized, according to the severity of the COPD. In addition, to evaluate the results, simple tests associated with the physical performance of the lower limbs, previously validated in COPD, have been used, such as the four-meter walk, speed test (4MGS) and the five-repetition test sitting and standing (5STS). The objective was to evaluate whether the Individualized Non-Presential Exercise Training PrOgram (NIETO) induces improvements in the 4MGS, 5STS and quadriceps muscle strength (QMS) tests in outpatients with advanced COPD (FEV1 ≤ 50%). After one year, the QMS was significantly higher in the intervention group (IG) than in the control group (CG) (2.44 ± 4.07 vs. 0.05 ± 4.26 kg; *p* = 0.009). The 4MGS and 5STS tests were significantly shorter in IG than in CG (−0.39 ± 0.86 vs. 0.37 ± 0.96 s; *p* = 0.001) and (−1.55 ± 2.83 vs. 0.60 ± 2.06 s; *p* = 0.001), respectively. A home model of simple muscle training monitored by telephone such as NIETO, can improve 4MGS, 5STS, and quadriceps strength tests in outpatients with advanced COPD.

## 1. Introduction

Patients with chronic obstructive pulmonary disease (COPD) usually have reduced daily physical activity [1,2] and subjects who perform some level of regular physical activity have a lower risk of both COPD admissions and mortality [3]. Low physical activity is a strong risk factor of hospitalization and mortality among patients with COPD [4,5,6]. Therefore, a key objective in the management of COPD in clinical practice is to improve the level of physical activity and exercise capacity [7,8,9,10].

However, many patients with COPD have impaired peripheral skeletal muscle strength and mobility activity limitations [11,12]. Limb muscle dysfunction is a clinically relevant systemic manifestation of COPD because it influences important clinical outcomes in this disease [13,14]. Up to a third of COPD patients, even in early stages of their disease, show impaired muscle function in their limbs and strength 25% lower than that developed by control subjects [15]. The reduction of muscle strength is more easily demonstrable in the lower extremities of patients with COPD [16]. Consequently, a primary focus of medical treatment is to detect these dysfunctions and develop specific and effective rehabilitation strategies [7,8,9,10,17].

Pulmonary rehabilitation (PR) has been shown to be the most effective non-pharmacological intervention for improving health status in COPD patients and has become a standard of care for COPD patients [7,9]. One aim of PR in patients with COPD is to increase the level of physical activity and to reduce functional limitations by improving physical performance [7,8,9,10,18,19]. Many PR programs have been developed and provided by multidisciplinary teams and typically include components such as patient assessment, exercise training, education, nutritional intervention, and psychosocial support [7,8,9,10]. Unfortunately, the real-life use of RP in COPD is practically non-existent or lacking in resources in most countries [19,20]. Many factors contribute to the gap between the evidence on the benefits of PR and the effective delivery of these services. Among the most commonly described problems are accessibility, low adherence, loss of benefits over time and costs [20]. In this context, in recent years, RP interventions have been implemented at home, which overcome the limitations of the traditional RP model, center-based [21]. These non-face-to-face RP interventions are less expensive [22] and aim to overcome barriers to patient access to traditional programs to improve adherence in patients [20]. The elimination of healthcare barriers and simplicity are two of the strengths of the home model, and no statistically or clinically significant differences are found for the results of exercise capacity or health-related quality of life between home RP versus ambulatory [20,21,22].

With this same orientation of applicability and simplicity, compared to more complex tests to determine exercise capacity, such as the six-minute walk test (6MWT) [23]; in recent years have been developing and validating faster and more simple tests in COPD, such as the Short Physical Performance Battery (SPPB) and two of its three subtests: the four-meter walk speed test (4MGS) and the five-meter -repetition sit-to-stand test (5STS) [24,25,26]. The 4MGS or 5STS is plausible that could replace the 6MWT in this type of evaluation [25,26]. In addition, they have recently been identified as potentially useful markers for classifying subjects with poor performance of the 6MWT [27,28]. SPPB has been suggested to be a valid measure to identify patients with deficiencies in quadriceps strength [29]. Because lower extremity strength is important for the successful completion of these mobility activities, SPPB has also been cited as a measure of lower extremity physical performance and a discriminatory tool for identifying COPD patients at risk for disability [30,31]. Also, these tests are commonly used to evaluate physical performance in other populations with lower-limb impairments [24].

Nevertheless, few studies have used simple exercise tests for evaluating physical performance following pulmonary rehabilitation in patients with COPD [32]. To our knowledge, there are no studies investigating the effectiveness of non-presential individualized exercise training in lower limb physical performance in patients with COPD using these simple exercise tests.

Thus, this study aims to assess whether Non-presential Individualized Exercise Training PrOgram (NIETO) induces benefits in the 4MGS, 5STS tests and quadriceps muscle strength (QMS) in outpatients with advanced COPD who had severe or very severe functional impairment, in stable clinical.

## 2. Materials and Methods

### 2.1. Study Design and Participants

In this quasi-experimental study, patients with severe COPD were prospectively recruited from an outpatient pulmonary department at Morales Meseguer General Universitary Hospital in Murcia, Spain. The study protocol was approved by the institutional review board of the hospital, called the “Ethical Committee of Clinical Research of the General University Hospital” (approval number: EST-43/15). The recruitment period was June to December 2016. All study participants provided written informed consent.

Over a 1-year period, recruited patients enrolled on NIETO. The conditions for inclusion in the Intervention Group were:

- Advanced COPD: all the patients included had severe respiratory functional impairment: FEV1≤ 50% or very severe: ≤30%; of the reference values; according to the recommendations of the Global COPD Initiative (GOLD), a postbronchodilator ratio of forced expiratory volume in one second (FEV1)/forced vital capacity postbronchodilator <70% and FEV1 <50% predicted [7].
- Stable COPD: all the patients when they were included in the study were COPD in a situation of clinical stability, outside a period of exacerbation or hospitalization (six weeks prior to inclusion)

Patients were excluded when they had an unstable cardiac condition; cognitive deterioration; or were unable to perform exercise training. Fifty-eight patients were included in the Intervention Group (IG).

Comparison Group (CG) was created from a database of 137 outpatients with stable COPD by statistical matching technique by sociodemographic, clinical, pulmonary and non-pulmonary variables and followed during the same time [31].

### 2.2. Non-Presential Individualized Exercise Training PrOgram Description

**Figure 1:**
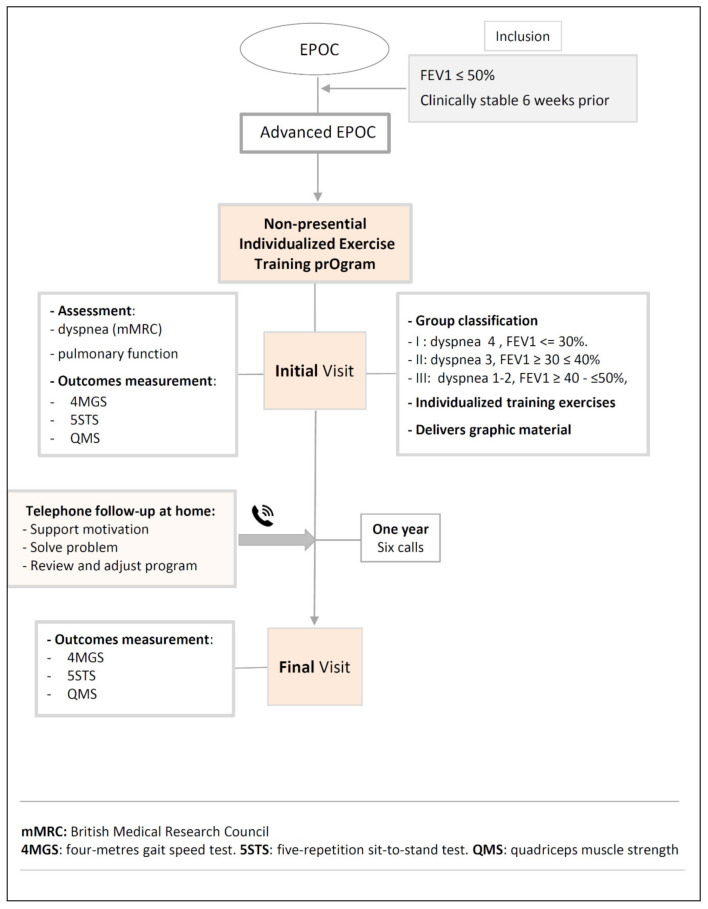
Systematic Non-presential Individualized Exercise Training prOgram (NIETO).

**Figure 2:**
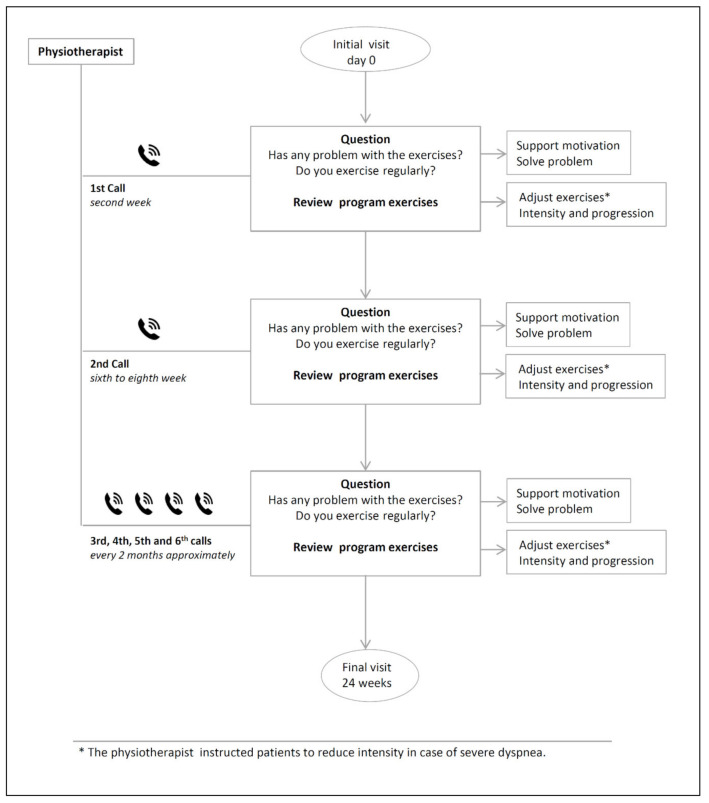
Description of Algorithm Telephone follow-up at home.

**Figure 3:**
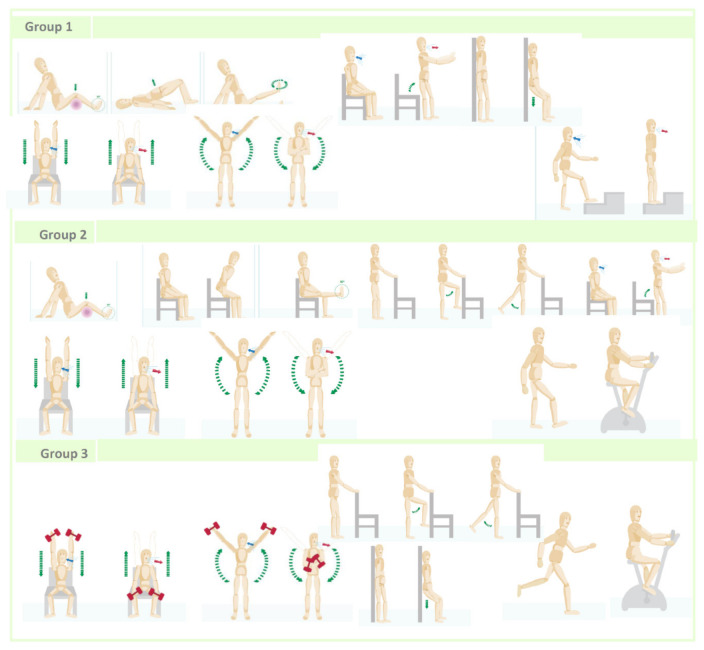
Individualized exercise training for each group of NIETO.

The program was individualized because each of patients was evaluated according to symptoms and functional status, received personalized training on the exercises that should be carried out at home, according to these characteristics and was followed by phone for a year to resolve problems, find out about her adherence and make incremental changes to her exercise program. During the face-to-face visit of the program, a physiotherapist from the pulmonology service evaluated the IG patients and classified them into three groups. The classification of the patients into was related according to the level of dyspnea (mMRC scale) and functional deterioration in spirometry, according to the fact that all patients had a decrease in FEV1 less than 50% of the reference value. Group I included patients with dyspnea 4 and FEV1 ≤ 30%. The Group II: dyspnea 3 and FEV1 ≥ 30 and ≤40% and Group III 1–2 and FEV1≥ 40 and ≤ 50%. Each patient, according to the assigned group, was taught resistance and aerobic exercises, an incremental exercise routine was programmed in terms of intensity (low, moderate and high) and repetitions. The individualized exercise training session lasted 20 to 30 min. At the end, graphic material was delivered with a detailed explanation of the weekly exercise program (Annex 1). All patients enrolled in NIETO were followed up by telephone. The first call was made at two weeks and the second between the sixth and eighth weeks and then every 2 months until the final visit, at 24 weeks. In the calls, the physiotherapist asked about the existence of problems, adherence to the exercises, solving doubts and encouraging him to continue; finally, the intensity and progression in exercises was reviewed and adjusted. The description of the NIETO program systematics, exercise programming (type, intensity and duration), telephone follow-up and graphic material delivered to the patients is developed in Figure 1, Figure 2 and Figure 3 and Supplementary Table S1.

### 2.3. Primary Outcome Measurement

The primary outcome measurement was lower limb physical performance using 5STS test, 4MGS test and QMS. The primary outcome was measured at baseline and at the year following.

Participants performed the SPPB according to the National Institute on Aging protocol [24]. The times of its 3 components: standing balance, 4MGS test, and five-repetition sit-to-stand test (5STS) were measured with a stopwatch. The 5STS test measured the time (seconds) taken to stand 5 times from a sitting position, as rapidly as possible. The 4MGS test measured the time (seconds) taken to cross a 4-m line at a typical walking speed [24].

Isometric QMS was evaluated following standard manual muscle-testing procedures [33]. A hand-held dynamometer was used to improve the objectivity of the force estimates (Nicholas Manual Muscle Tester, model 01160, Lafayette Instrument Company, Lafayette, IN, USA; for quadriceps) To measure isometric QMS, participants stayed seated with their knees flexed 70 degrees. The dynamometer was placed on the anterior tibia, 5 cm above the lateral malleolus, and a break test was performed [33,34].

### 2.4. Other Variables

The sociodemographic variables included age (years) and sex. Clinical and pulmonary variables included the body mass index (BMI, kg/m^2^) obtained by measuring the weight (kg) and height (m); number of comorbidities measured using the CO-morbidity TEst (COTE) [35]; depression, assessed by the depression subscale of the Hospital Anxiety and Depression Scale (HAD-D), with probable depression considered when the total score was ≥11 [36]; forced expiratory volume in 1 s (FEV_1_), measured with post-bronchodilator spirometry (MasterScope Spirometer, version 4.6, Jaeger, Würzburg, Germany), according to the American Thoracic Society guidelines [7]; dyspnea, measured with the self-administered modified British Medical Research Council (mMRC) [37]; and the patient’s medical history was reviewed to determine smoking pack-years and current smoker status.

### 2.5. Statistical Analysis

Descriptive data are expressed as the mean and standard deviation (SD) or number and percentage of the total sample. Paired Students’ *t* tests were used to evaluate post-intervention changes at group level. Control Group (CG) was created by statistical matching technique by clinical variables [38]. The significance level was set at *p* < 0.05. Holl corrections was used control Type I. Type I errors might occur due to the different number of test done. Cohen’s d was calculated to estimate the effect size of changes in QMS, 4MGS and 5STS tests [39]. Effect size was calculated using an online calculator [40]. Sample size calculation was established for examining statistical significance by the *t*-test and using QMS as the main outcome measure. The sample size was based on 80% power to detect an effect size of 0.55 between groups, assuming an alpha level of 0.05. A total of 42 participants per group were required.

All analyses were performed using the Statistical Package for the Social Sciences (SPSS) version 24.0 (IBM SPSS, Chicago, IL, USA).

## 3. Results

Participants

A total of 58 patients were included, of which 43 completed the NIETO program (74.1%). Of the 15 lost patients (25.1%), 3 did not repeatedly answer calls, one patient died due to non-respiratory causes, one patient underwent surgery and abandoned the program, the remaining ten patients (17.2%) acknowledged that they did not continue exercises regularly. Finally, 43 patients with advanced COPD who completed the NIETO were analyzed Compared to patients in the CG, patients of the IG were similar in relation to the baseline characteristics (*p* > 0.05). The patients’ baseline characteristics are shown in Table 1.

At baseline, the intervention cohort had a mean age of 67 years; most subjects were male (79.1%), had a mean FEV_1_ of 40% predicted value and 16.3% were current smokers. A non-significant difference in the QMS, 4MGS test and 5STS test was observed in the IG versus the CG (*p* > 0.05).

At 1-year following, the QMS was significantly greater in IG than in the CG (2.44 ± 4.07 vs. 0.05 ± 4.26 kg; *p* = 0.009). The 4MGS test decreased 0.39 ± 0.86 s in the IG while this test increased 0.37± 0.96 s in the CG (*p* = 0.001). The 5STS test decreased 1.55 ± 2.83 s in the IG while this test increased 0.60 ± 2.06 s in the CG (*p* = 0.001). In IG, the effect size (ES) and 95% confidence interval (CI) for the QMS was 0.57 (95% CI = 0.142, 1.005), the ES for the 4MGS test was 0.02 (95% CI = −0.401, 0.445), and the ES for the 5STS test was 0.38 (95% CI = −0.043, 0.810). Data of the effect size in the QMS, 4MGS and 5STS tests and the change between initial and final visit from the CG and IG are shown in Table 2.

Effect size and change in the quadriceps strength, 4MGS and 5STS tests at 1 year following, according to belong to the intervention group (IG) or the control group (CG)

Table 3 shows the results of the ES and 95% confidence interval (CI) of QMS, 4MGS and 5STS tests categorised by gender, smoking, dyspnea, FEV_1_ and depression. In the IG, the ES and 95% CI of quadriceps strength of male, non-smoking, with dyspnea ≤ 2, FEV1 < 30% predicted value and HAD-D ≥ 11, was 0.725 (0.234, 1.216), 0.834 (0.342, 1.327), 1.116 (0.431, 1.800), 0.711 (0.241, 1.181) and 0.487 (−0.044, 1.018), respectively. The ES of 4MGS and 5STS tests across subgroups of IG and CG are shown in Table 3.

**Figure 4:**
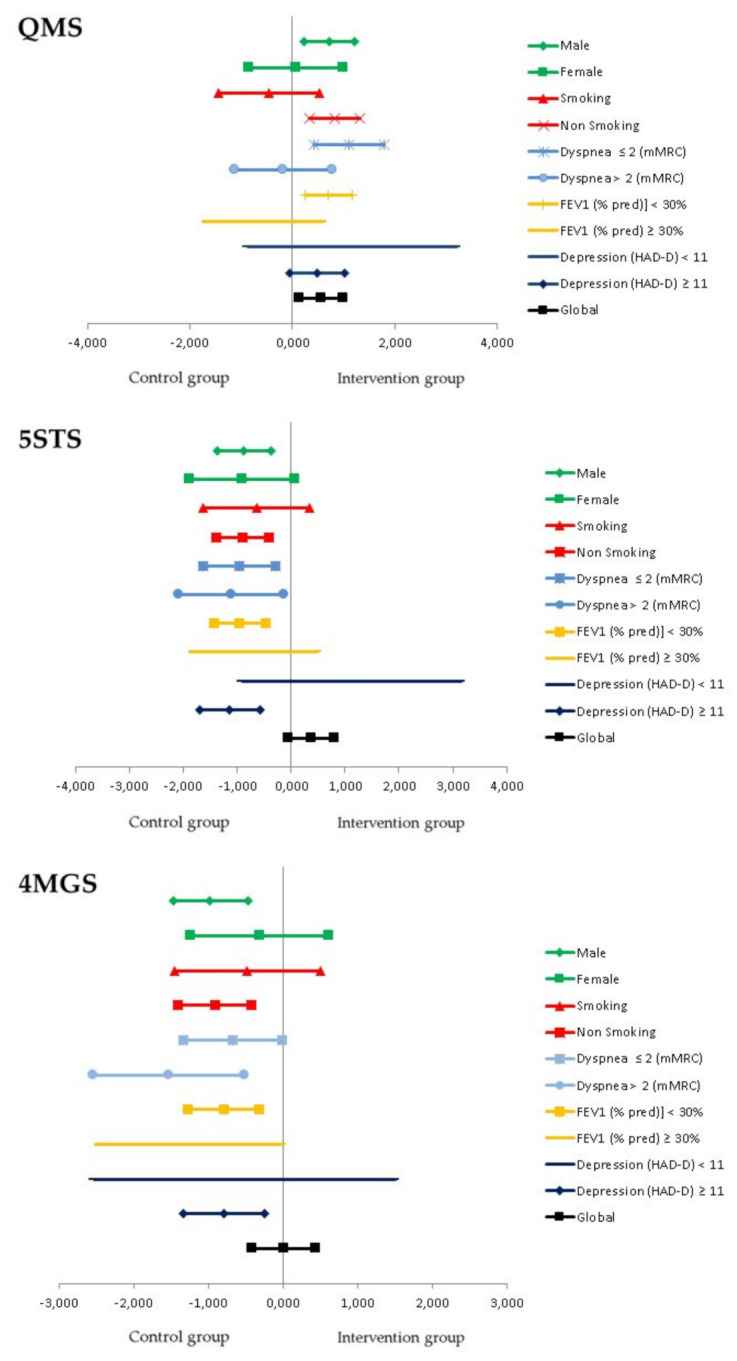
Forest plot of effect size and 95% confidence interval of quadriceps muscle strength (QMS), five-sit-to-stand test (5STS) and 4-metre gait-speed test (4MGS) categorized by gender, smoking, dyspnea, FEV1 and depression, according to belong to the intervention (IG) or control group (CG).

Forest plots shown in Figure 4 showed differences in the effect size of QMS, 5STS and 4MGS tests across subgroups of patients with COPD enrolled in NIETO. When analyzing QMS categorized by gender, smoking, dyspnea, FEV1 and depression of the IG, the ES (95% IC) in male, non-smoking, dyspnea ≤ 2, FEV1 < 30% predicted value and HAD-D ≥11, was 0.725 (0.234–1.216), 0.834 (0.342–1.327), 1.116 (0.431–1.800), 0.711 (0.241–1.181) and 0.487 (−0.044–1.018), respectively.

## 4. Discussion

In this study, we assessed whether *Non-presential Individualized Exercise Training PrOgram (NIETO)* induces benefits in the 4MGS, 5STS tests and quadriceps strength at the following year among outpatients with severe stable COPD. The 4MGS and 5STS tests were significantly faster and the quadriceps strength was significantly greater in patients enrolled in NIETO. In addition, the 4MGS and 5STS tests may be simple tools for evaluating the enhancement in lower limb physical performance among patients with COPD after the application of exercise training.

It is well documented that exercise training improves physical performance in COPD [7,8,9,41,42]. Despite the positive effects, fewer than 10% of people with COPD engage in traditional outpatient PR [19,20]. In an attempt to increase the participation rate, some facilities offer personalized home-based PR, especially appropriate for the more severe patients [20,21,22]. In COPD, home-based PR is feasible and carries the same benefits in the short and long term as the inpatient or outpatient program [20,21]. A systematic review of 12 clinical trials, up to 2013 comparing pulmonary vs. home rehabilitation, did not find, statistically or clinically significant differences for the results of exercise capacity or related quality of life, but low quality of the studies analyzed [21].

Nevertheless, some clinical trials in de home-based rehabilitation for COPPD using minimal resources produced short-term clinical outcomes that were equivalent to center-based pulmonary rehabilitation have shown benefits in functional exercise capacity. A trial with 252 patients con COPD (Mean FEV1% predicted; SD: 43; 13), after a 4-week educational program, were randomized to home rehabilitation or outpatient hospital rehabilitation for 8 weeks and followed for 1 year during the follow-up, a visit was scheduled at three months and they were followed up by telephone every 4 weeks to identify adverse events. The results in dyspnea, quality of life and tolerance to exercise measured by the six-minute walk test were equivalent in both models [43]. In our study, the COPD population with similar functional impairment (mean FEV1% predicted ± SD: 40 ± 11) received an educational program in a single session and only two face-to-face visits were made during the follow-up year. In addition, the telephone follow-up was carried out with the aim of encouraging patients to be adherents and adjust the performance of exercises.

The use of telephones as a tool for the application and supervision of an exercise training program for patients with moderate to severe COPD in a home environment, has been used by different studies with good clinical and compliance results [44]. However, in a recent meta-analysis, the evidence for effectiveness in physical function was inconsistent across studies and the combined effect size was not significant with notable variation in the outcome measures used between trials [45]. Most PR studies in COPD that compare home versus hospital-based programs have analyzed outcomes in quality of life or exercise capacity (6MWT) [21,43,46], but few studies have focused on the effect of home RP interventions on muscular strength or functional performance of lower limbs. The study by Chen et al. [47] compared the effects of a home-based PR of six sets of lower limb training for 12 weeks, while the control group received routine PR guidance only. The outcome measures were muscle strength and functional status by isokinetic/isometric extensor muscle peak torque (PT) and five-repetition sit-to-stand test. Unlike our study, the 25 patients in the intervention group had better lung function (FEV1% predict: 54.49 ± 23.62), the intervention was more intensive and included trips to the patients’ homes, and the results were measured within a week twelve.

Another interesting aspect of NIETO is the supervision of patients for one year, although we do not know the effect of this component of the program. That most studies were conducted as remotely supervised, such as via home visits or telephone calls by a physiotherapist or doctor [46,48], although this aspect has been little analyzed, some studies indicate that unsupervised outpatient programs in patients with COPD are less effective [49], although in general there is a lack of consensus in the design and standardization of these interventions [46,49].

Several previous studies have utilised the usual gait speed as an outcome measure, particularly in patients who have received rehabilitation or exercise interventions after recent hospitalization, hip fracture, spinal cord injury or stroke [50,51]. In our study, the 4MGS and 5STS improved significantly, and had a medium and large effect size, respectively [39]. These results are consistent with previous reports [32,52,53].

In our study, among subgroups of patients with COPD enrolled in NIETO, the isometric QMS improved significantly in male, non-smoking patients with dyspnea ≤2 and FEV1 < 30% predicted value. Quadriceps strength was also greater in patients with depression (HAD-D ≥ 11). Patel et al. [29] have previously reported quadriceps strength to be predictive of SPPB scores. As quadriceps muscle dysfunction is common [11,12,13,14] and predicts mortality in COPD [54], it is encouraging to find that NIETO may improve QMS and the 4MGS and 5STS tests.

As for the SPPB subtests, in this study, there was no change in the SPPB balance subtest, but the 4MGS and 5STS improved significantly with NIETO, probably balance subtest the balance component of the SPPB may be explained by the fact that successful balance maintenance is a complex skill that also relies on other determiners, such as the integration and coordination of musculoskeletal systems (i.e., biomechanics, range of motion, and flexibility) and neural systems (i.e., motor, sensory, and higher-level premotor processes) and few patients in our study showed balance impairments. Further, the subscales of the SPPB do not provide equivalent information regarding the status of a COPD patient. The observed similarities and differences between subscales of the SPPB in previous studies have a variety of implications in various situations in which one test might be selected. For example, if a clinician wants a screening tool to detect poor health status in COPD patients, the 5STS would appear to be the most suitable test. If a test is needed to assess outcome measures following an intervention aimed at improving psychological status, the 4MGS would be an exceptionally good choice. On the other hand, if an intervention relates to exercise tolerance and/or QMS, the 5STS test may be a more suitable functional outcome tool [55].

However, the 5STS is reliable, valid and responsive in patients with COPD with an estimated minimal clinically important difference (MCID) of 1.7 s [53]. Kon et al. recently reported the test–retest and interobserver reliability and concurrent validity of the 4MGS in patients with COPD, finding that the 4MGS improves with pulmonary rehabilitation and declines with time in COPD, with an estimated MCID of 0.11 m.s^−1^ [56]. A significant advantage of the 4MGS and 5STS is simplicity. Assessment time is typically 1–2 min and the tests require only a stopwatch. The 4MGS is also readily acceptable to patients as walking is a familiar activity and the test may be more indicative of activities of daily living and is easy to understand and the short distance may relieve any anxieties associated with having to complete a maximal or near-maximal test. Furthermore, both tests require little space, which means the 4MGS and 5STS test could be adopted in most clinical environments. In comparison, well-established field walking tests in COPD, such as the 6MWT or the shuttle walk, required more space and time which can limit their widespread use in some clinical environments. Therefore, the 4MGS and 5STS test are a practical functional outcome measurement suitable for use in most healthcare settings.

The most recent American Thoracic Society/European Respiratory Society Policy Statement on Pulmonary Rehabilitation identified increasing the accessibility of pulmonary rehabilitation as a key priority [8]. For home-based programs to generate benefits equivalent to the current “gold standard” for center-based pulmonary rehabilitation, they must be easy to implement in cost and complexity, both in their development and in the use of outcome measurement tools. Our study has been focused on these principles, although we have not calculated the costs of NIETO, similar studies indicate economic savings while improving the accessibility of patients to RP [57].

### 4.1. Implications for Practice and Research

Although the traditional center-based model of RP in COPD is very effective in clinical trials [58], there is poor adherence in clinical practice. While some barriers reflect inefficiencies in the health system and low referral rates, there are also patient-related barriers to program acceptance. These include travel and transportation to the rehab center; disease and comorbidities; inconvenient time; and disruption of established routines [20]. Home-based programs have the potential to overcome many of these limitations. It should be recognized that it is easier to “attend” a phone call at home than a rehabilitation session in the hospital. In the current socio-sanitary context, as a result of the COVID-19 pandemic, many pulmonary rehabilitation programs have transitioned rapidly to remote delivery models [59,60] at the same time that simpler tests are incorporated that can also be performed at home, as used in this study, 4MGS and 5STS tests [61].

Despite the fact that the application of SPPB requires more time than the application of any of its components alone, it has been widely recommended because it could provide a more complete assessment of the physical capacity of the lower extremities [24,32]. Our study suggests that the 4MGS and 5STS tests alone could be sufficient to assess the physical performance of the lower extremities at home, using fewer resources than other hospital tests such as the 6-minute walk test.

Other studies, in patients and different healthcare settings, are necessary to replicate and confirm the benefits of the Program (NIETO) with the tests used (4MGS, 5STS and QMS). The elimination of healthcare barriers and simplicity have been objectives of our study, and therefore we consider that our proposal for non-face-to-face intervention and the results measurement tests are the strengths of the program.

### 4.2. Strengths and Limitations of the Study

This work has some strengths. In the first place, the present study extends previous studies that demonstrated that an individualized physical training program away from the classroom is feasible and has benefits in patients with advanced COPD. Second, it is a prospective quasi-experimental study with a control group created from a database of 137 outpatients with COPD with the same functional impairment, using a statistical matching technique and followed up during the same period (1 year). Another strength, we believe, has been the individualized approach in the scheduling of trials and the systematic telephone follow-up of each case. There are also obvious advantages to the 4MGS and 5STS as easy, inexpensive and rapid assessment tools, validated in COPD, with no “learning effect” detected [53]; making them feasible in most healthcare settings, including the home. In contrast, the tools most used so far in COPD to evaluate the effect of RP, like 6MWT, require more time and resources [61].

This study also had some limitations. First, since it is not multicenter, its results must be generalized with caution. Future prospective studies with different populations and centers are needed to confirm these results. Second, some variables were not controlled, especially the performance of other RP interventions in the control group. Third, the adherence of patients to the program, in terms of how many times per week or for how long, was not objectively measured and it is plausible to think that there were different ranges of compliance among the patients in the intervention group. Fourth, the information resulting from the phone calls was not systematically collected, in terms of how many problems the patients had or the difficulties in implementing the program.

Regarding the tests used, the main limitation of the 5STS test is the presence of a “floor” effect and this test may have an increased value in patients with better functioning compared to patients who cannot attempt or complete the test [53]. Other simple functional outcome tests, such as typical walking speed of more than 4 m, may be more appropriate for more poorly functioning individuals [32,52,53]. Our study also highlighted a limitation associated with the SPPB balance subtest, namely a ‘ceiling’ effect, although this effect does not affect the 4MGS and 5STS subtest scores as outcome measures tools [52,53]. Finally, a more rigorous evaluation of the feasibility of the program should have included a global study of costs per patient, including the performance of outcome evaluation tests.

## 5. Conclusions

In conclusion, in the current COVID-19 pandemic context, health systems have decided to implement virtual care approaches that reduce, as far as possible, the need for physical meetings between patients and health providers. Traveling to a medical center receive training, can be a significant barrier for many patients and cause additional logistical difficulties to those already existing. For this reason, the NIETO program acquires special relevance due to its home approach and because it shows a positive effect on the performance of the lower limbs in a population of patients with advanced COPD with severe physical limitations. New prospective studies should confirm these findings, with similar approaches and simple assessment tools, incorporating necessary aspects such as the cost-effectiveness of home-based RP programs [62,63,64].

## Figures and Tables

**Table 1:** Baseline characteristics of the study population (intervention and control group).

Characteristics	Intervention Group (IG) *n* = 43	Control Group (CG) *n* = 43	*p*-Value
**Sociodemographic variables**			
Age (years), mean (SD)	67.83 (9.49)	67.04 (9.32)	0.698
Female, n (%)	9 (20.9)	9 (20.9)	1.000
**Clinical and Pulmonary variables**			
BMI (kg/m^2^), mean (SD)	27.47 (4.65)	28.89 (4.80)	0.167
COTE, mean (SD) Depression (HAD-D), mean (SD) FEV_1_ (% predicted), mean (SD) Current smoker, n (%) Dyspnea (mMRC), mean (SD)	1.5 (2) 11.46 (7.82) 40.34 (11.89) 7 (16.3) 2.83 (0.68)	0.8 (1.6) 13 (8) 44.09 (13.09) 10 (23.3) 2.51 (0.85)	0.383 0.169 0.417 0.678
**Non-pulmonary variables**			
Quadriceps strength (kg), mean (SD)	17.35 (4.03)	15.83 (2.92)	0.188
4MGS (seconds), mean (SD)	4.73 (1.20)	4.39 (1.00)	0.329
5STS (seconds), media (SD)	17.24 (4.33)	14.30 (2.73)	0.139

Abbreviations: SD, standard deviation; BMI, body mass index; Kg, kilograms; m, meters; COTE, CO-morbidity Test; HAD, Hospital Anxiety and Depression Scale; FEV1, forced expiratory volume in 1 s; m MRC, modified British Medical Research Council; 4MGS, 4-m gait-speed test; 5STS, five-sit-to-stand test.

**Table 2:** Effect size and change in the quadriceps muscle strength, 4MGS and 5STS tests at 1 year following, according to belong to the intervention group (IG) or control group (CG) ^a^.

Outcome Measure	Intervention Group (IG) *n* = 43	Control Group (CG) *n* = 43	*p*−Value	Effect Size (95% CI)
Quadriceps strength (kg), mean (SD)	2.44 (4.07)	0.05 (4.26)	0.009 *	0.57 (0.142, 1.005)
4MGS (seconds), mean (SD)	−0.39 (0.86)	0.37 (0.96)	0.001 *	0.02 (−0.401, 0.445)
5STS (seconds), mean (SD)	−1.55 (2.83)	0.60 (2.06)	0.001 *	0.38 (−0.043, 0.810)

Abbreviations: Intervention Group, IG; Control Group, CG; CI, confidence interval; Kg, kilograms; SD, standard deviation; 4MGS, 4-metre gait-speed test; 5STS, five-sit-to-stand test. ^a^ Values represent the mean change (measurement difference between the last and first visit) * *p* < 0.05.

**Table 3:** Baseline, mean change and mean difference at 1 year following in quadriceps muscle strength, 4MGS and 5STS tests categorised by gender, smoking, dyspnea, FEV_1_ and depression, according to belong to the intervention (IG) or control group (CG).

	**Baseline**		**Mean Change at 1 Year**		**Mean Difference** **(95% CI)**
	**IG**	**CG**	**IG**	**CG**	
	**Quadriceps Muscle Strength (Kg)**				
**Gender**					
Male	20.21 (4.99)	15.64 (3.23)	2.47 (3.86)	−0.45 (4.21)	−2.92 (−4.886, −0.970) *
Female	18.25 (5.54)	16.93 (3.15)	2.32 (5.02)	1.95 (4.13)	−0.370 (−4.969, 4.229)
**Smoking**					
Current	15.15 (4.12)	18.12 (2.34)	0.44 (3.99)	2.21 (3.79)	1.76 (−2.300, 5.836)
No current	20.70 (4.81)	15.21 (3.17)	2.83(4.02)	−0.60(4.23)	−3.43 (−5.421, −1.452) *
**Dyspnea (mMRC)**					
≤2	21.56 (4.56)	15.97 (3.16)	4.31 (3.74)	−0.25 (4.19)	−4.572 (−7.300, −1.843) *
>2	19.11 (5.20)	15.59 (4.01)	1.72 (4.01)	2.41 (4.46)	0.689 (−3.299, 4.678)
**FEV_1_ (% pred)**					
<30%	17.01 (4.78)	17.66 (3.23)	2.755 (4.12)	−0.279 (4.39)	−3.034 (−5.013, −1.055) *
≥30%	20.34 (5.04)	15.73 (3.21)	0.842 (3.62)	2.560 (1.76)	−0.569 (−1.739, 0.601)
**HAD−D**					
<11	18.70 (1.65)	18.70 (0.67)	2.815 (4.78)	−2.800 (0)	−5.615 (−15.789, 4.557)
≥11	19.82 (5.16)	15.96 (3.44)	2.053 (3.23)	0.118 (4.29)	−1.935 (−4.060, 0.189)
	**Baseline**		**Mean Change at 1 year**		**Mean Difference** **(95% CI)**
	**IG**	**CG**	**IG**	**CG**	
	**4MGS (Seconds)**				
**Gender**					
Male	4.45 (1.17)	4.70 (1.36)	−0.45 (0.87)	0.44 (0.97)	0.899 (0.451, 1.347)
Female	3.95 (1.12)	4.40 (1.18)	−0.19 (0.85)	0.07 (0.91)	0.270 (−0.614, 1.154)
**Smoking**					
Current	4.68 (0.82)	4.68 (1.03)	−0.16 (1.18)	0.25 (0.59)	0.419 (−0.504, 1.342)
No current	4.28 (1.22)	4.62 (1.41)	−0.44 (0.80)	0.40 (1.05)	0.848 (0.399, 1.296)
**Dyspnea (mMRC)**					
≤2	4.23 (1.41)	4.58 (1.37)	−0.32 (0.82)	0.29 (0.96)	0.623 (0.001, 1.244)
>2	4.39 (1.09)	5.15 (0.35)	−0.42 (0.89)	0.92 (0.84)	1.351 (0.481, 2.221)
**FEV_1_ (% pred)**					
<30%	5.38 (1.58)	4.60 (0.41)	−0.41 (0.88)	0.33 (1.00)	0.751 (0.313, 1.189)
≥30%	4.14 (0.97)	4.63 (1.36)	−0.29 (0.84)	0.65 (0.58)	0.942 (−0.036, 1.922)
**HAD−D**					
<11	4.57 (2.60)	5.73 (1.06)	−0.38 (0.66)	−0.05 (0)	0.337 (−1.079, 1.753)
≥11	4.34 (1.18)	4.44 (0.89)	−0.40 (1.05)	0.37 (0.97)	0.788 (0.254, 1.323)
	**Baseline**		**Mean Change at 1 year**		**Mean Difference** **(95% CI)**
	**IG**	**CG**	**IG**	**CG**	
	**5STS (Seconds)**				
**Gender**					
Male	17.31 (4.39)	14.01 (2.33)	−1.66 (3.07)	0.67 (2.20)	2.340 (1.046, 3.633)
Female	15.53 (4.31)	13.88 (3.85)	−1.12 (1.75)	0.34 (1.48)	1.466 (−0.155, 3.089)
**Smoking**					
Current	18.30 (3.71)	14.77 (2.91)	−0.30 (1.06)	0.68 (1.77)	0.981 (−0.625, 2.587)
No current	16.91 (4.46)	13.69 (2.63)	−1.79 (3.01)	0.58 (2.16)	2.377 (1.107, 3.648)
**Dyspnea (mMRC)**					
≤2	14.99 (2.22)	13.90 (2.77)	−1.59 (2.26)	0.45 (2.09)	2.058 (0.637, 3.479)
>2	18.01 (4.69)	15.38 (0.72)	−1.53 (3.06)	1.71 (1.50)	3.244 (0.382, 6.106)
**FEV_1_ (% pred)**					
<30%	17.58 (6.60)	13.63 (1.97)	−1.86 (2.81)	0.44 (1.95)	2.304 (1.185, 3.423)
≥30%	17.07 (3.97)	14.01 (2.79)	0.04 (2.53)	1.84 (2.66)	1.801 (−1.572, 5.174)
**HAD−D**					
<11	17.01 (1.97)	13.78 (0.80)	−1.09 (2.51)	−3.94 (0)	−2.841 (−8.195, 2.512)
≥11	17.15 (4.39)	14.02 (2.86)	−2.02 (3.12)	0.71 (1.95)	2.741 (1.457, 4.026)

Abbreviations: Intervention Group, IG; Control Group, CG; CI, confidence interval; Kg, kilograms; SD, standard deviation; mMRC, modified British Medical Research Council; FEV_1_, forced expiratory volume in 1 s; HAD-D, depression subscale of the Hospital Anxiety and Depression Scale; 4MGS, 4-metre gait-speed test; 5STS, five-sit-to-stand test. * *p* < 0.05.

## Data Availability

The data presented in this study are available on request from the Juan Miguel Sánchez-Nieto.

## Supplementary Materials

The following are available online at https://www.mdpi.com/2077-0383/10/5/1010/s1, Table S1: Exercise programming (type, intensity and duration).
