# Supplementary material for: Effectiveness of Non-Presential Individualized Exercise Training PrOgram (NIETO) in Lower Limb Physical Performance in Advanced COPD
Source: J Clin Med. 2021 Mar 2;10(5):1010. doi: 10.3390/jcm10051010 (PMC7958336; doi:10.3390/jcm10051010)
Supplement: Supplementary file 1 [file jcm-10-01010-s001.pdf]

Program with Individualized Non-Presential Exercise the Physical Performance of the Lower Extremities

Exercise Program

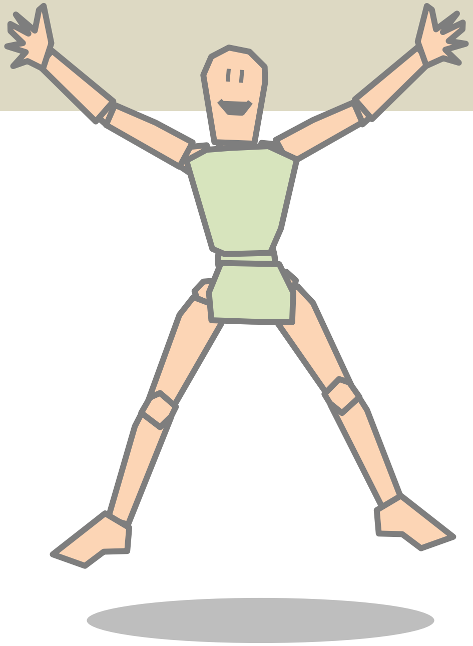

to do at home

Advises and General Information: Physical Exercise

To practice a full physical exercise it must be worked:

Breathe normally while exercising, without holding your breath at any time.

Muscle training:

- *Exercise intensity standards (Repetitions)*
- *Appropiate training frequency (3-7 days a week)*
- *Duration (mínimum 20 min. Session)*

Aerobic exercise:

- Minimum three time per week during twenty minutes.
- Continuous way (All time and distance at same rate).
- Intervals (dividing time in total distance, having breaks in movement and changing rates).

It is better to take inhalers before starting to improve performance and exercise tolerance.
In case of using home oxygen, use it during exercise.
Keep normal breathing during exercise. Do not hold breath.

Physical Exercise: Legs

Grupo 1

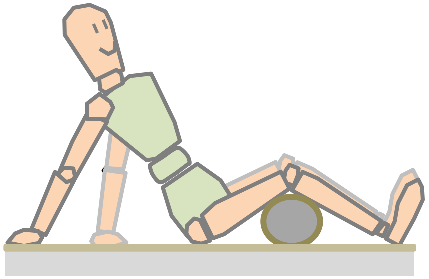

Sitting on the floor, stretch legs and straight back, set a roller(rolled towel) under your knees and keep ankle at 90º. Pressing towel.

Number of repetitions

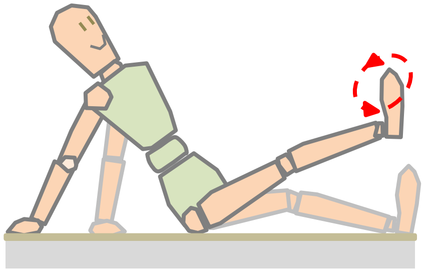

Lying down with one stretched leg and the other one flexed. First lifting one leg full stretched 30 cm. from the ground, in this position draw little circles as we were drawing with the ankle. Repeat with both legs.

Number of repetitions

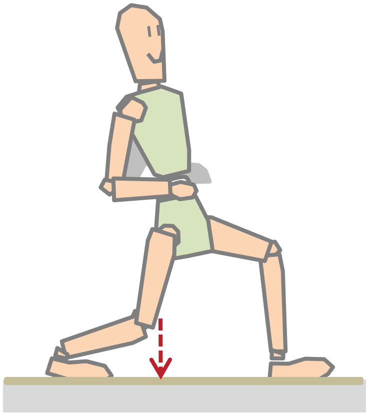

Upright, legs lightly separated from the hip. Breathe and take a step keeping straight trunk. Forwarded leg must flex till thigh, set as much parallel to ground as posible. Rear leg stays fixed by foot to the ground but it must lift down to foot till knee. Exhaling back to initial position.

Number of repetitions

Physical Exercise: Legs

Grup 1

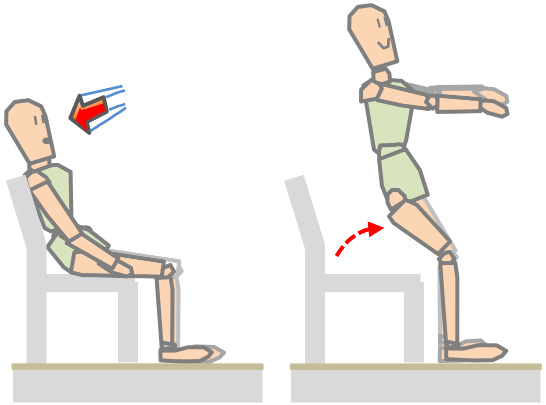

From sitting position, feet on the ground, inhale and stand up, no leaning on. Full breathing and back to initial position.

Number of repetitions

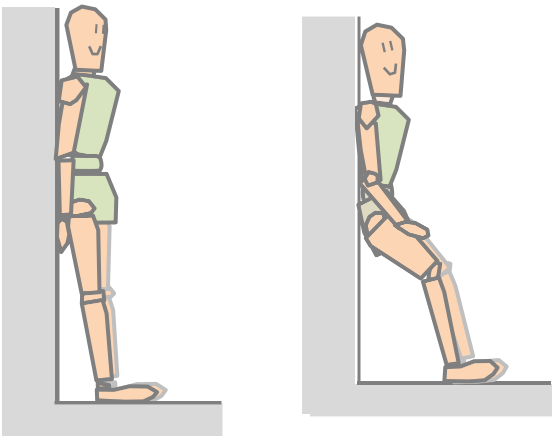

Leaning back and buttocks on the wall, parallel and separated feet, go slowly down flexing legs and keeping the back on the wall till possible. Then go up to initial position without leaning on.

Number of repetitions

Physical Exercise: Legs

Grup 1

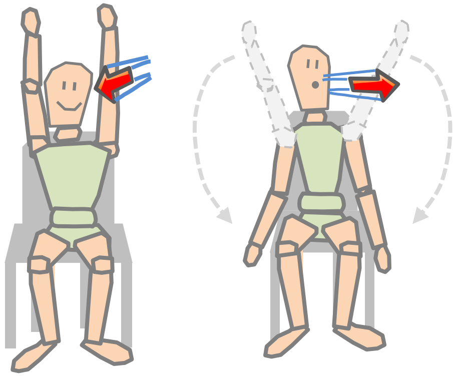

Sitting down on a chair, feet on the ground.
Inhale while arms up to maximum, ahead of eyes.
Exhale slowly, lips frowning, arms down to initial position.
Standing up is optional.

Number of repetitions

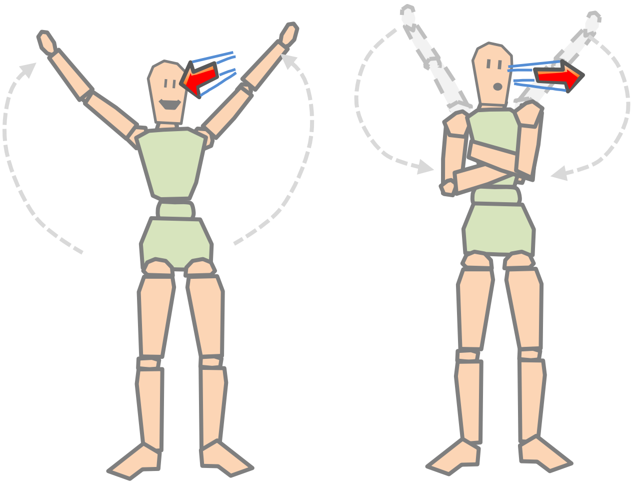

Inhale while opening arms to máximum and exhale slowly wiht tight lips, hugging oneself. Sitting down is optional.

Number of repetitions

Physical Exercise: Legs

Grup 1

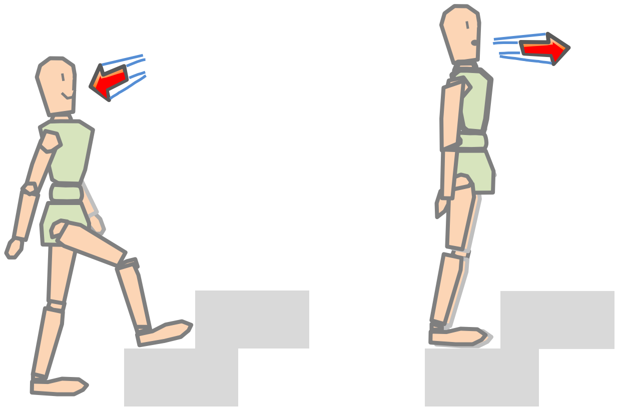

Climb a step inhaling, stop and exhale.
Climb another step inhaling and climb two steps more exhaling.
Repeat.

Number of repetitions

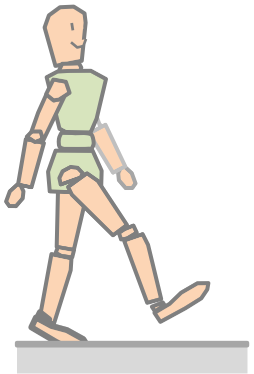

Slow walking on flat ground, no strain. If one feels fatigue, stop, recover and keep walking.
Avoid the warmest and the coolest moments of the day

week									
1	2	3	4	5	6	7	8	9	10
**minuts**									
15	20	25	30	35	40	45	50	55	60

Physical Exercise: Legs

Grup 2

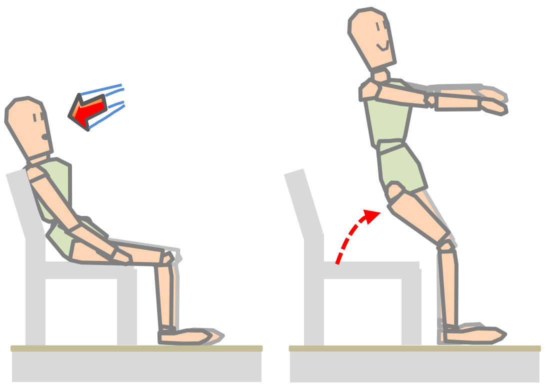

From sitting position, feet on the ground, inhale and stand up, no leaning on. Full breathing and back to initial position.

Number of repetitions

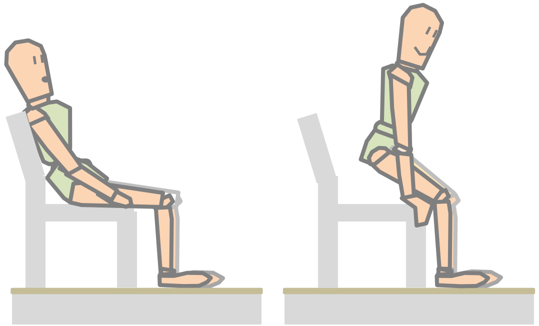

From sitting position, feet on the ground, leaning hands at the edge of the chair.

Lift bottom up as one wanted to stand up, but did not. Make effort with arms but specially with buttocks and legs.

Number of repetitions

Physical Exercise: Legs

Grup 2

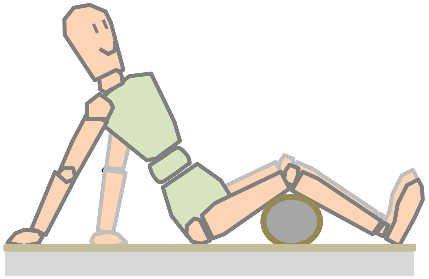

Sitting on the floor, stretch legs and straight back, set a roller(rolled towel) under your knees and keep ankle at 90º. Pressing towel.

Number of repetitions

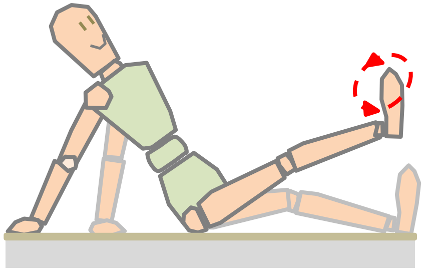

Lying down with one stretched leg and the other one flexed. First lifting one leg full stretched 30 cm from the ground, in this position draw little circles as we were drawing with the ankle. Repeat with both legs.

Number of repetitions

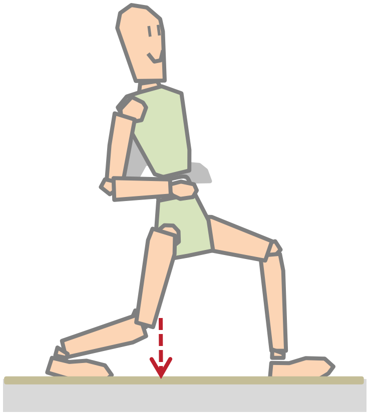

Upright, legs lightly separated from the hip. Breathe and take a step keeping straight trunk. Forwarded leg must flex till thigh, set as much parallel to ground as posible. Rear leg stays fixed by foot to the ground but it must lift down to foot till knee. Exhaling back to initial position.

Number of repetitions

Physical Exercise: Arms

Grup 2

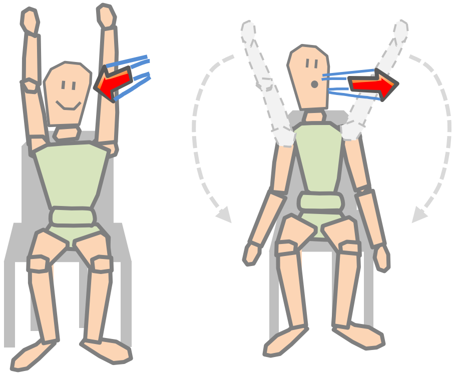

Sitting down on a chair, feet on the ground.
Inhale while arms up to maximum, ahead of eyes.
Exhale slowly, lips frowning, arms down to initial position. Standing up is optional.

Number of repetitions

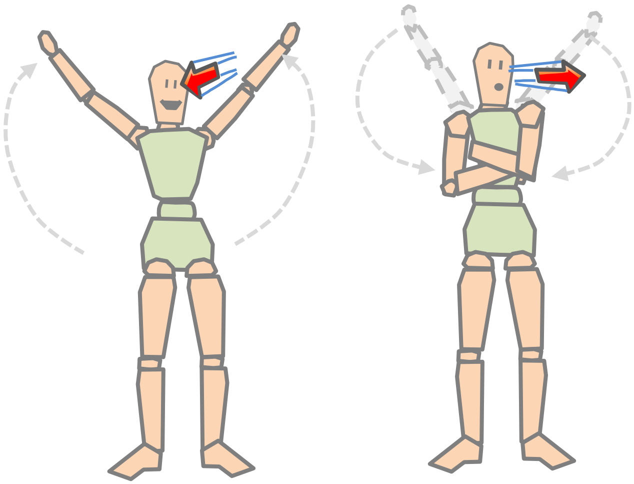

Inhale while opening arms to máximum and exhale slowly wiht tight lips, hugging oneself.
Sitting down is optional.

Number of repetitions

*Start doing the exercises as explained above and we are adding weight little by little and according to our tolerance

Physical Exercise: Walking, bicycle, stairs

Grupo 2

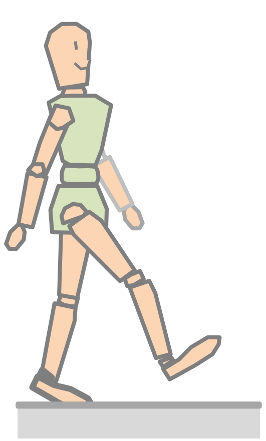

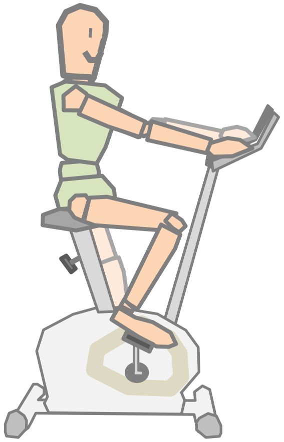

Slow walking on flat ground, no strain. If one feels fatigue, stop, recover and keep walking.
Avoid the warmest and the coolest moments of the day

**wek**									
1	2	3	4	5	6	7	8	9	10

**minuts**							
15	20	25	30	35	40	45	50

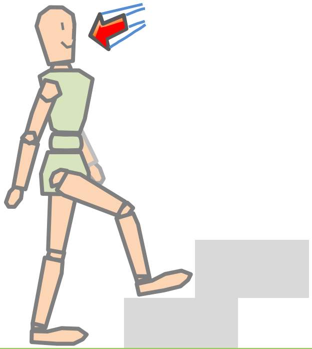

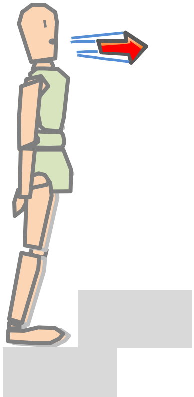

Climb a step inhaling, stop and exhale.
Climb another step inhaling and climb two steps more exhaling.
Repeat.

Number of repetitions

Physical Exercise: Legs

Grup 3

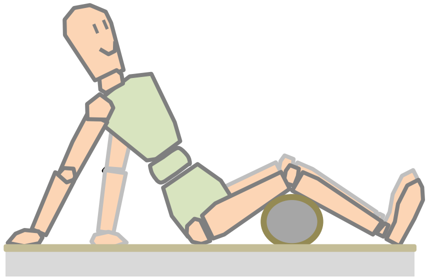

Sitting on the floor, stretch legs and straight back, set a roller(rolled towel) under your knees and keep ankle at 90º. Pressing towel.

Number of repetitions

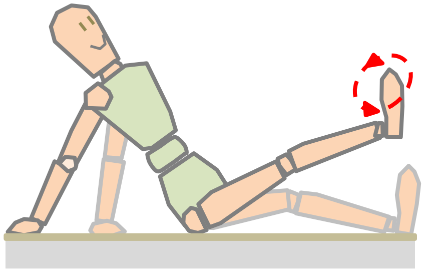

Lying down with one stretched leg and the other one flexed. First lifting one leg full stretched 30 cm. from the ground, in this position draw little circles as we were drawing with the ankle. Repeat with both legs.

Number of repetitions

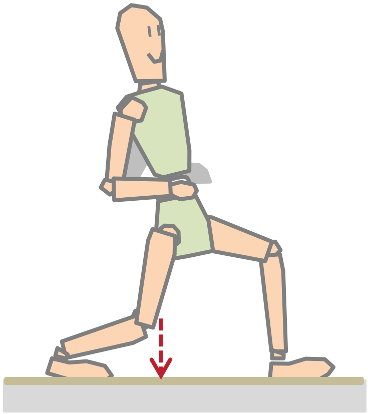

Upright, legs lightly separated from the hip. Breathe and take a step keeping straight trunk. Forwarded leg must flex till thigh, set as much parallel to ground as posible. Rear leg stays fixed by foot to the ground but it must lift down to foot till knee. Exhaling back to initial position.

Number of repetitions

Physical Exercise: Legs

Grup 3

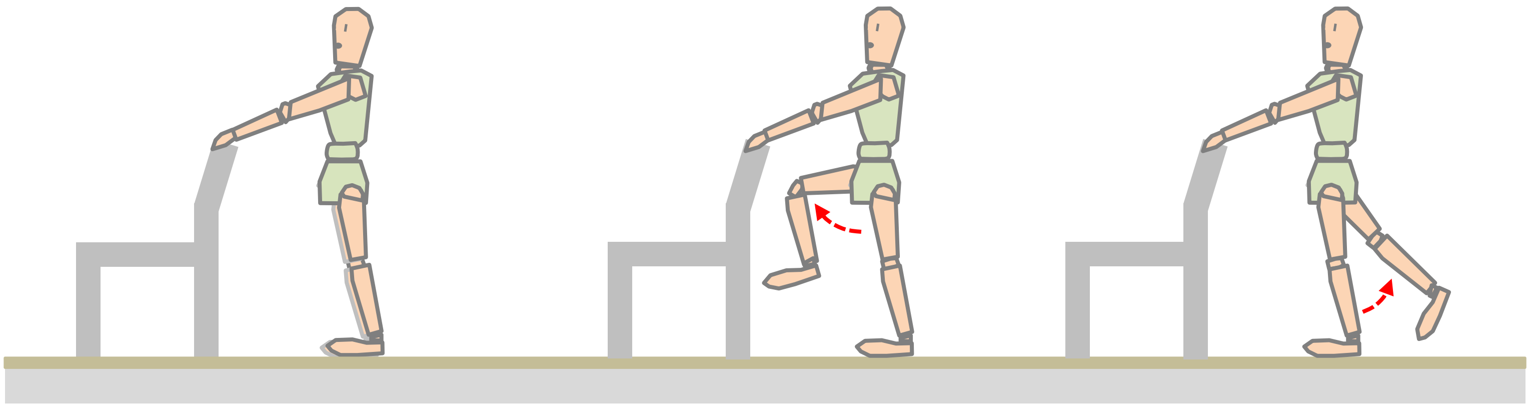

Standing, support your arms on a fixed chair. Bend one knee (about 90^0 degrees), then extend it and bring it back about 30^0 degrees, without touching the ground. It is important to keep the body straight during exercise.

Number of repetitions

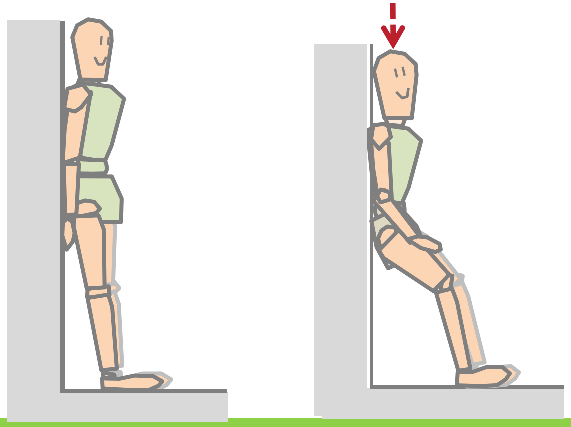

Leaning back and buttocks on the wall, parallel and separated feet, go slowly down flexing legs and keeping the back on the wall till possible. Then go up to initial position without leaning on.

Number of repetitions

Physical Exercise: Arms

Grup 3

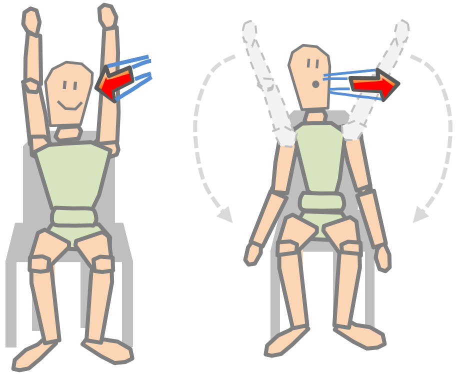

Sit in a chair with feet flat on the floor
Take in air as we raise our arms to the maximum, in front of our eyes, breathe out slowly with tight lips, slowly lower our arms to the starting position. This exercise can be done standing

Number of repetitions

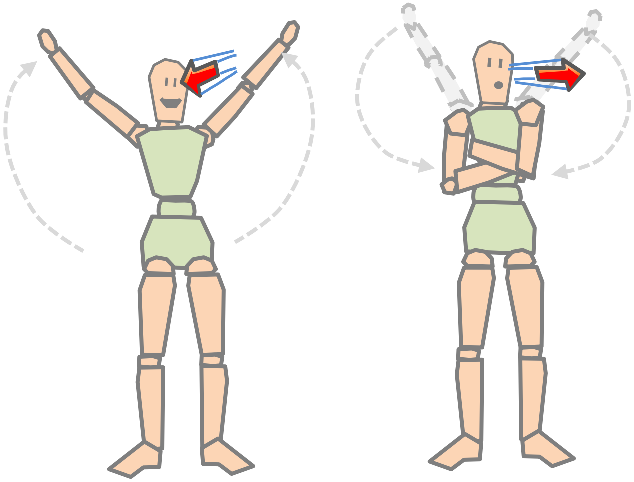

Take in air while we open our arms to the maximum and breathe out slowly with tight lips, hugging ourselves. This exercise can be done sitting down.

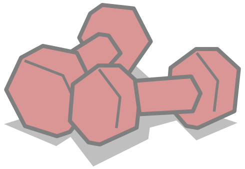

Number of repetitions

It can be done with half a kilo of weight in each hand, and progressively increase according to tolerance.

Ejercicios: **Andar, bicicleta, correr**

Grupo 3

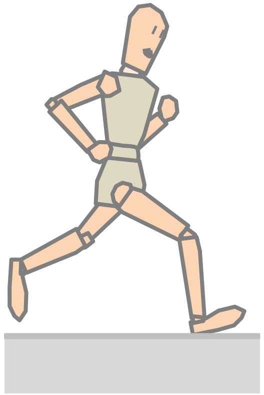

run or walk quickly slowly, on flat terrain and without forcing, increasing according to difficulty breathing, at the pace you tolerate, when you feel fatigue, stop, recover and continue slower

Avoid the warmest and the coolest moments of the day

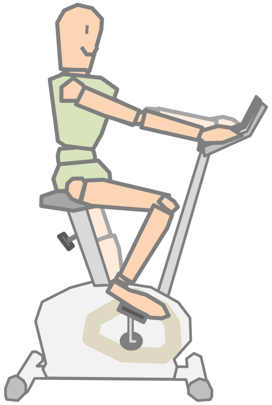

If it is an exercise bike, increase the resistance little by little according to the respiratory difficulty, do not forcé.

**wek**									
1	2	3	4	5	6	7	8	9	10

**minuts**							
15	20	25	30	35	40	45	50
